# CO_2_ Capture and Release in Amine Solutions: To What Extent Can Molecular Simulations Help Understand the Trends?

**DOI:** 10.3390/molecules28186447

**Published:** 2023-09-05

**Authors:** Changru Ma, Fabio Pietrucci, Wanda Andreoni

**Affiliations:** 1 Institute of Physics, Ecole Polytechnique Fédérale de Lausanne, 1015 Lausanne, Switzerland; 2 Sorbonne Université, Muséum National d’Histoire Naturelle, UMR CNRS 7590, Institut de Minéralogie, de Physique des Matériaux et de Cosmochimie, IMPMC, F-75005 Paris, France

**Keywords:** carbon dioxide capture, aqueous amine solutions, reaction dynamics in solution, molecular dynamics simulations

## Abstract

Absorption in amine solutions is a well-established advanced technology for CO_2_ capture. However, the fundamental aspects of the chemical reactions occurring in solution still appear to be unclear. Our previous investigation of aqueous monoethanolamine (MEA) and 2-amino-2-methyl-1,3-propanediol (AMPD), based on ab initio molecular dynamics simulations aided with metadynamics, provided new insights into the reaction mechanisms leading to CO_2_ capture and release with carbamate formation and dissociation. In particular, the role of water—strongly underestimated in previous computational studies—was established as essential in determining the development of all relevant reactions. In this article, we apply the same simulation protocol to other relevant primary amines, namely, a sterically hindered amine (2-amino-2-methyl-1-propanol (AMP)) and an aromatic amine (benzylamine (BZA)). We also discuss the case of CO_2_ capture with the formation of bicarbonate. New information is thus obtained that extends our understanding. However, quantitative predictions obtained using molecular simulations suffer from several methodological problems, and comparison among different chemical species is especially demanding. We clarify these problems further with a discussion of previous attempts to explain the different behaviors of AMP and MEA using other types of models and computations.

## 1. Introduction

In the search to mitigate the harmful effects of global warming, carbon capture and storage play a key role. In particular, post-combustion capture (PCC) is considered the most viable approach for achieving the zero-carbon goal (see, e.g., [1,2]). Currently, the most mature post-combustion technology relies on CO_2_ absorption into amine solutions (see e.g., [3]). Several complementary or different PCC technologies are under investigation, either to enhance the performance of amine solutions, e.g., by adding a catalyst (see e.g., [4,5,6]), or as alternatives. The latter is based, for example, on membrane contactors (see e.g., [7]), calcium looping (see e.g., [8]), or CO_2_ hydrates [9]. Numerous assessments of techno-economic aspects have been performed (for recent publications see, e.g., [1,10,11]).

This paper focuses on the capture and release of CO_2_ in amine solutions and, in particular, on the fundamental properties of these systems. As is well-known, several critical challenges have not been addressed in the use of aqueous amines, mainly regarding degradation and energy consumption (see, e.g., [12,13]). The prototype solution is aqueous monoethanolamine (MEA), which has become the benchmark for assessing new capturing agents and is the subject of numerous experimental and theoretical studies. Easy availability, high absorption efficiency, high amine solubility in water, and low price are among its well-known advantages, whereas proneness to both thermal and oxidative degradation and a high energy requirement for regeneration are among its disadvantages. Several other primary, secondary, and tertiary amines have been explored. Sterically hindered amines, of which 2-amine-2-methyl-1-propanol (AMP) is a remarkable example, have been widely investigated because they provide a relatively high loading capacity and a low energy requirement for regeneration, albeit they have a relatively low absorption efficiency (see e.g., [14,15,16]). The most successful strategy for an optimized amine-based capturing agent consists of blending two different amines. In this way, one can better exploit the fundamental properties of each amine, namely, the relatively high absorption rate of one and the relatively low regeneration energy penalty of the other.

The performance of different amines is in part associated with the nature of the predominant products of the absorption process, which can be detected experimentally with diverse probes (NMR, IR, VLE…): either carbamate or bicarbonate. Indeed, carbamate corresponds to a faster absorption rate than bicarbonate and also has a higher energy requirement for the regeneration process. The prevalence of one or the other product is strongly amine-dependent and is not easily/completely understood. Indeed, the reaction mechanisms leading to both the uptake and the release of CO_2_ elude experiment.

However, several suggestions have emerged from both experimental and theoretical approaches. Steric hindrance—due to bulky substituents adjacent to the nitrogen in the amino group, e.g., as in AMP vs. MEA—has long been recognized as an intrinsic source of carbamate instability, due to, for example, the interaction between methyl groups and the carboxylate group [14]. Hence, one deduces that bicarbonate would result from the hydrolysis of the CO_2_ released from carbamate. Further destabilization of the carbamate of sterically hindered amines is believed to derive from the reduced exposure to the solvent due to the presence of bulky substituents [16]. On the other hand, the latter might also reduce the accessibility to the amine group for CO_2_ molecules, thus reducing the tendency to form a carbamate. The availability of bases in solution is essential for the formation of both carbamate and bicarbonate. Therefore, the basicity of an amine itself is considered important, especially for carbamate formation, and is influenced by the molecular structure, depending on the presence of either electron-donating or electron-withdrawing groups at the a-carbon [17]. However, its role in the performance of the amine solutions is not obvious. For example, it appears to affect the behavior of the equilibrium vapor pressure as a function of loading in a different way for different amines [14], whereas a plot of the loading capacity (considering 76 amines) shows only a weak dependence on the pK_a_ for both primary and secondary amines [18]. Moreover, NMR measurements obtained from several primary and secondary amine solutions, including MEA and AMP, have shown that the amount of carbamate measured at equilibrium does not correlate with the relative basicity. However, it appears to correlate well with the ^15^N chemical shift, which is a measure of the electron density at the nitrogen [19].

Carbamate formation is observed in aromatic amines [20]. In principle, a comparison of their behavior with that observed in MEA could help to better understand the influence of the molecular structure on the mechanisms of CO_2_ absorption and desorption. In Ref. [21], the similarity between benzylamine (BZA) and MEA is emphasized, especially regarding the stability of the carbamate. However, in agreement with previous observations [22], the authors point out a major disadvantage of BZA, namely, the occurrence of precipitation at low temperatures and low CO_2_ partial pressures. To our knowledge, computational studies are lacking.

Over the past few years, using DFT-based molecular dynamics (MD) simulations, we studied the mechanisms leading to the formation and dissociation of carbamates in aqueous MEA [23,24] and 2-amine-2-methyl-1,3-propanediol (AMPD) [25], which is a partially sterically hindered amine. Our calculations showed that the role of water, which until then had been greatly underestimated, is crucial in determining both the reaction paths and energetics. We also revealed that the interaction between free amines, protonated amines, carbamates, and water significantly affects vibrational properties, which are used for the speciation of the solution after CO_2_ uptake [26].

In this paper, we apply the same methods to 2-amine-2-methyl-1-propanol (AMP) and benzylamine (BZA) solutions and compare them to MEA. We focus on the issue of carbamate formation and decomposition. We also consider the formation of carbamic acid and that of bicarbonate.

Our aim is to contribute to the identification and understanding of significant trends and also to critically point out the limitations of previous as well as our own computational approaches. Indeed, representing chemical reactions in a single amine solution is challenging [27], and comparing different amines involves further obstacles for a quantitative assessment.

Following the seminal papers by da Silva and Svendsen [28,29,30,31], several computational approaches appeared in the literature that also aimed at understanding trends in CO_2_ absorption by different aqueous amines. In general, these studies are based on a quantum chemical description of the amine and derived molecules and mainly (but not only) on a continuum polarisable model for the water solvent. It is not the purpose of this paper to provide an overview of this research; instead, we refer the reader to recent reviews [32,33,34]. However, we shall report the results of certain recent studies that can be compared with ours and also separately provide a brief description of the methods there used.

## 2. Results and Discussion

In this Section, we shall report our results for the capture of CO_2_ into AMP and BZA solutions. First, we focus on absorption via carbamate formation and on the comparison with our previous findings for MEA and AMPD. Then, we discuss absorption via bicarbonate formation and present our simulations in MEA and AMP solutions. Prior to the investigation of the chemical reactions of interest, we examine the structural properties of the amine molecules in the gas phase and in solution.

### 2.1. The Systems: Structural Properties

#### 2.1.1. AMP

**Figure 1:**
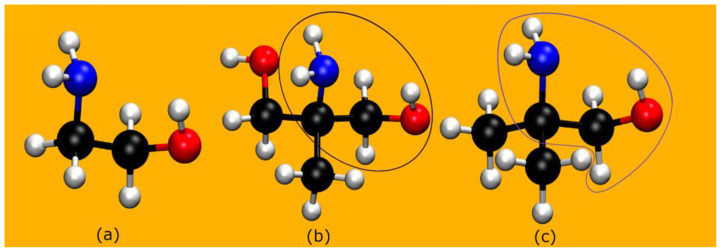
A comparison among (**a**) MEA, (**b**) AMPD, and (**c**) AMP. The MEA unit is encircled. Color code for the spheres: white = hydrogen; black = carbon; blue = nitrogen; red = oxygen.

As shown in Figure 1, both AMPD and AMP molecules can easily be considered as derived from MEA via substitution of the hydrogens at the α-carbon: with one methyl and one hydroxymethyl group in AMPD and two methyl groups in AMP. Given that the α-carbon is bound to three other carbon atoms (tertiary carbon), both these MEA derivatives are classified as sterically hindered amines [14]. As first pointed out in Ref. [14], steric hindrance to binding a CO_2_ molecule induces important differences onto the reactivity of AMP relative to MEA.

**Figure 2:**
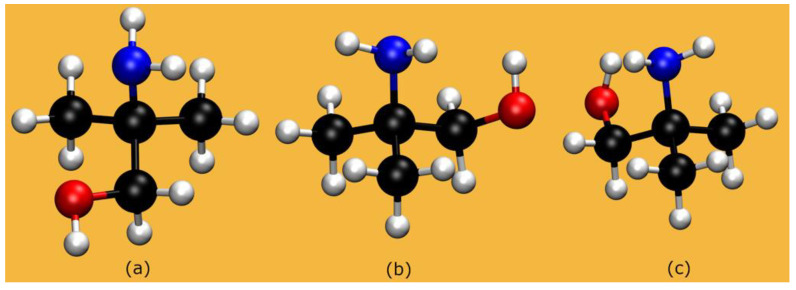
AMP: The three isomers considered in our calculations.

Our simulations are applied to the three different AMP isomers illustrated in Figure 2. The dihedral angles of the MEA-like moiety are shown in Table 1 for the isolated molecules (see Table S1 for comparison with all-electron calculations) and in solution.

#### 2.1.2. BZA

**Figure 3:**
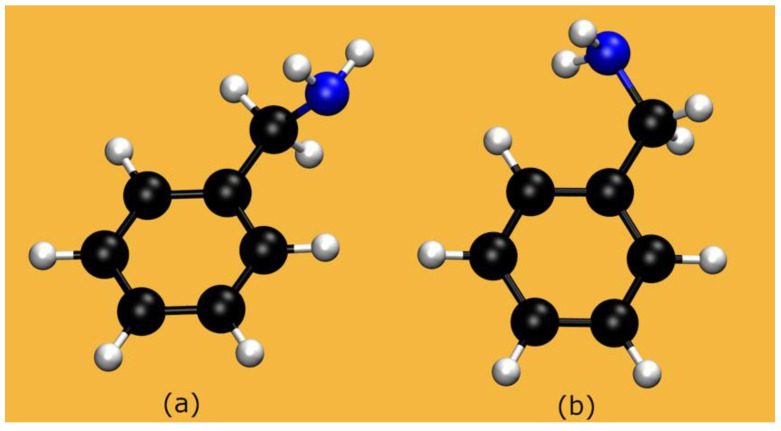
BZA: Both isomers considered in our calculations.

Figure 3 shows the two conformers of BZA considered here. The dihedral angles are listed in Table 2, for the isolated molecules (see Table S2 for a comparison among all-electron calculations) and in solution.

### 2.2. Reactions: CO_2_ Uptake and Release via Carbamate

The global chemical reaction leading to the capture of CO_2_ in an amine aqueous solution via carbamate is generally expressed as

CO_2_ + 2RNH_2_ ↔ RNHCOO^−^ + RNH_3_^+^ (1)

where the radical R can be written as CH_2_(CH_2_OH) for MEA; as (CH_2_OH)_2_CH_3_ for AMPD; as C(CH_2_OH)(CH_3_)_2_ for AMP and as C_6_H_5_CH_2_ for BZA.

According to the most common interpretation, the forward reaction in Equation (1) proceeds with the formation of a zwitterion

CO_2_ + RNH_2_ → RNH_2_^+^COO^−^ (2a)

followed by deprotonation and formation of a protonated amine:

RNH_2_^+^COO^−^ + RNH_2_ → RNHCOO^−^ + RNH_3_^+^ (2b)

Carbamate formation is known to be accompanied by the creation of protonated amines. Although other bases that can take up the proton are present in a solution, namely, water molecules and free hydroxyl ions, the most probable ultimate product of the deprotonation of the zwitterion is a protonated amine. Our models for the reactant do not include free hydroxyl ions.

The presence of carbamic acid (RNHCOOH) observed in some experiments also suggested an alternative mechanism (see e.g., [18,35]), where carbamic acid plays the role of the zwitterion in Equation (2a). As reported in [25], our own simulations of AMPD revealed a path that could also lead to the formation of carbamic acid, namely, where the zwitterion transfers a proton to an oxygen in the carboxylate anion via a proton wire:

RNH_2_^+^COO^−^ → RNHCOOH (3a)

Carbamate would then result from deprotonation of the carbamic acid:

RNHCOOH + RNH_2_ → RNHCOO^−^ + RNH_3_^+^ (3b)

The reverse reaction in Equation (1) is generally considered to be the process responsible for the dissociation of the carbamate. In Ref. [24], based on our estimates of the free-energy barriers corresponding to several reactions in MEA, we proposed that the most probable process leading to the regeneration of the amine and the release of CO_2_ again involves the zwitterion:

RNH_2_^+^COO^−^ → CO_2_ + RNH_2_ (4)

This reaction is the ultimate step in a three-step mechanism, namely, it follows the deprotonation of an RNH_3_^+^ and the protonation of the carbamate (reverse of Equations (2a) and (2b)).

Our simulations of the above reactions in AMP and BZA solutions followed the same protocol established in our previous studies [24,25]. In the following, we report and compare our estimates for the FEBs relative to the various reactions here studied. These precise values are affected by statistical uncertainty (about 2 kcal/mol) and are systematically overestimated by at least 3 kcal/mol errors. We provide the lowest FEB calculated for the different isomers. Note, however, that the latter differ by 2 kcal/mol at most.

#### 2.2.1. AMP

The search for carbamates after CO_2_ capture in AMP solutions has eluded most experimental tools. Only analysis of ^13^C NMR data on a 30 wt.% solution was able to detect the presence of AMP carbamates for loadings ranging from 0.12 to 0.62 [36,37].

Using the C-N distance between the carbon in CO_2_ and the nitrogen in an amine as the collective variable, metadynamics simulations were run for each of the three AMP isomers in the 30 wt.% solution, thus leading to a zwitterion as in Equation (2a). However, in all cases, when the metadynamics bias was switched off, the zwitterion did not survive and spontaneously released the CO_2_ moiety (as in Equation (4)). This behavior is in sharp contrast with our results for both MEA and AMPD and appears to be consistent with the absence of AMP-carbamate in most experimental reports.

However, a carbamate can still form in the real systems, e.g., through processes [38] or conditions other than those represented in our model. Thus, we built carbamate isomers from those of AMP (see Figure S1), which could be equilibrated in solution. The aqueous environment is different from MEA due to the presence of hydrophobic groups and, as pointed out in Ref. [17], plays a relatively weaker role in stabilizing the carbamate. This can be deduced when comparing the number and length of the hydrogen bonds between the carbamate and water molecules in AMP and the other amines (see Table 3).

Using the C-N distance as the collective variable, metadynamics-aided MD led to the release of CO_2_. We noticed that in the carbamate of the AMP-c isomer (Figure S1), an internal H-bond is present, which is expected to provide a relatively lower stability in solution [17]. However, this feature did not turn out to be relevant. In all three cases, the estimated FEBs were in the range of 18–20 kcal/mol. These values are significantly lower than those calculated in the other amines, namely, about 50 kcal/mol in both MEA and AMPD. The difference in the FEBs is thus consistent with the strength of the amine-COO bond, which electron-donating groups adjacent to nitrogen tend to weaken. In Ref. [24], our calculations showed that the reorganization of the solvent induced with the recovery of the CO_2_ molecule is accompanied by an important entropy contribution to the FEB (on the order of 10 kcal/mol) that facilitates the reaction. This is also the case in the AMP solution.

We recall that in the case of MEA solutions, stopped-flow kinetic measurements obtained at low concentration were interpreted in terms of a free-energy barrier of 15 kcal/mol for CO_2_ release, with negligible entropic contribution (0.3 kcal/mol) [39]. Therefore, as mentioned above, we proposed that in MEA, CO_2_ release proceeds via the zwitterion (Equation (4)), as the ultimate step of a reaction involving the deprotonation of an amine. The latter is the rate-limiting step and corresponds to an FEB of about 16 kcal/mol. The same value was obtained for the AMPH^+^ deprotonation. Therefore, in the case of AMP, carbamate dissociation via the protonated amine–zwitterion route appears to be competitive with the direct detachment. Also, our estimate of the enthalpic contribution to the FEB showed that the effect of the entropy change was negligible in both MEA and AMP.

We noticed that our results are in full contrast with the conclusions drawn in Ref. [40], suggesting that the instability of the AMP carbamate was related to the “shift of equilibrium between the carbamate and its zwitterion”.

Our findings also clearly disagree with those of Ref. [41]. In that study, the zwitterion was found to be a local minimum of the potential energy surface of the amine + CO_2_ system for both MEA and AMP, with energy barriers of about 12.9 and 17.8 kcal/mol, respectively. Deprotonation of the zwitterion was reported to correspond to 1.2 and 2.4 kcal/mol barriers, respectively. Both these values were beyond the accuracy attainable in such calculations and indeed only show that the event is spontaneous in both cases.

Indeed, the models in Refs. [40] and [41] (see the Methods Section below) are unable to represent the reactions of interest and, more generally, reactions that happen in an aqueous solution. These models not only ignore the crucial contributions of the solvent to the determination of the reaction paths and the energetics but they also completely neglect dynamical (finite temperature) effects. We refer the reader to our review of the computational work (up to 2016) on MEA [28], in which we clarify in detail the inadequacy of this type of approach.

Given the intrinsic limitations of the models studied in Ref. [42] (see the Methods Section), it is also difficult to reconcile their results with ours. Total energy calculations in explicit water did not qualitatively differ from those deduced from estimates of the relative (approximate) Gibbs free-energy differences in the molecules in implicit solvent; thus, both suggested that the carbamate was thermodynamically stable for MEA and AMP. Moreover, free ab initio molecular dynamics was run for 100 ps at 1000 K to investigate the behavior of the AMP carbamate. No sign of instability was observed.

#### 2.2.2. BZA

An experimental investigation revealed that BZA solutions behave similarly to MEA regarding CO_2_ capture, e.g., they show “similar reaction kinetics and carbamate stability” [22]. Similarity also emerges from our results and, in particular, from the values of the FEBs. In this case, both reactions (Equations (2a) and (2b)) leading to the formation of the carbamate are also not spontaneous: FEB values of about 14 kcal/mol and 10 kcal/mol correspond to the formation and deprotonation of the zwitterion, respectively. The values estimated in our models of aqueous MEA [24] were 14 kcal/mol and 8 kcal/mol, respectively. Therefore, within the intrinsic error in the calculations, we can consider those estimates indistinguishable. We also verified that the value of FEB did not change in a dilute solution (4.6 wt.%), in agreement with what we found in the case of MEA (3 wt.%).

**Figure 4:**
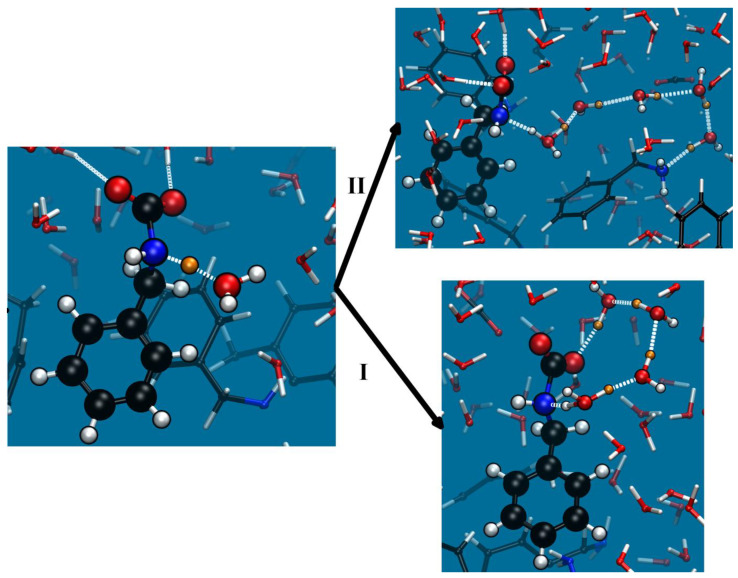
BZA: Snapshots showing deprotonation of the zwitterion (on the left) and two subsequent processes leading to the formation of either (**I**) carbamic acid (Equation (3a)) or (**II**) carbamate and a protonated amine (Equation (2b)). Color code as in Figure 1. The gold sphere represents a moving proton.

Also, in analogy with MEA, the BZA protonation proceeds via water molecules and is barrierless. As pointed out above, the formation of a carbamic acid (Equation (3a)) could act as a proton trap, slowing down the reaction, because its deprotonation is inhibited by an FEB of about 12 kcal/mol. Note that our estimate was about 8 kcal/mol in the case of MEA. However, one should also consider the relative probability for those two processes (Equation (2b) vs. Equation (3a)) to take place (see Figure 4).

As shown in a study of AMPD compared to MEA [25], at least in our models, relevant information could be inferred from the analysis of those specific simulations as follows. The proton released from the zwitterion reaches another amine or the carboxylate anion via a proton wire. Therefore, it is reasonable to assume that its fate depends to a certain extent on the hydrogen bond paths connecting the zwitterion to either the NH_2_ group in another amine or the COO^−^ group. Table 4 lists the average value of the length of the shortest H bond paths in both cases, which are also compared to MEA and AMPD. These data refer to free MD simulations of the systems before switching on the bias, and the averages are taken over 10ps. Clearly, these structural characteristics of the zwitterion environment do not favor the formation of carbamic acid in BZA, as in MEA. The different behavior observed for AMPD, namely, the formation of both the protonated amine and the carbamic acid as products of the zwitterion deprotonation, is consistent with Table 4. It was attributed [25] to the anisotropy of the water environment of the amine, which is due to the presence of a hydrophobic methyl group that reduces the access of a proton to other amines.

As noted in Ref. [24], our simulations showed that, beyond the two amines directly involved in the formation of carbamate, other amines can play a role in this reaction, in that they can participate in the chain of proton transfer events. On the contrary, they act as passive spectators in the release of CO_2_ from either carbamate or the zwitterion.

Regarding the BZA-carbamate (see Figure S2), Table 3 shows that its H-bond environment tends to stabilize it in solution, as in aqueous MEA. More specifically, in this case, the simulations showed that the presence of a hydrophobic ring leads water molecules to concentrate around the functional groups of the carbamate.

The characterization of the CO_2_ release provided the same results for the two amines. The estimated FEB for the direct detachment from the BZA-carbamate was about 47 kcal/mol, whereas the highest barrier involved in the amine BZAH^+^deprotonation-zwitterion path was found to be about 16 kcal/mol, which corresponds to the proton release into the solution.

By summing up the FEBs of both the forward and reverse reactions leading to CO_2_ capture and release via carbamate, we can estimate the free-energy difference between the amine solution with solvated CO_2_ molecules and the system containing one carbamate. As in the case of MEA [24], for the BZA, this difference is negligible within the accuracy of our calculations.

### 2.3. Reactions: CO_2_ Uptake via Bicarbonate

Dissolution of CO_2_ in water leads to the formation of carbonic acid (H_2_CO_3_) and bicarbonate (HCO_3_^−^) in the presence of hydroxyl ions [43]. HCO_3_^−^ is the most common product observed in amine solutions. In MEA, it is clearly detected for loadings higher than about 0.5 and is believed to derive from the decomposition of the carbamates [44]. In AMP, most speciation data obtained after capture can be interpreted only in terms of bicarbonate and protonated amines. Unlike the carbamate path (Equation (1)), CO_2_ capture via bicarbonate involves only one amine per CO_2_, thus leading to higher loading (capacity). The global reaction can be expressed as

CO_2_ + RNH_2_ + nH_2_O ↔ HCO_3_^−^ + RNH_3_^+^ + (n − 1) H_2_O, (5)

which involves the splitting of a water molecule: H_2_O ↔ H^+^ + OH^−^.

In this scenario, the difference between one amine and the other should mirror the relative ability to strip a proton from a water molecule, as expressed in the pK_a_ values of the conjugate acids. Experimental values at room temperature are 9.7 and 9.51 for AMP and MEA, respectively [28], so their difference corresponds to less than 1 kcal/mol for the difference in the dissociation Gibbs free energies of the conjugate acids. Therefore, the difference in the tendency to attract a proton from water cannot be accounted for in our calculations due to the insufficient statistical and quantum mechanical precision. Indeed, our estimates for the FEBs corresponding to bicarbonate formation are identical within the accuracy of our calculations, namely, about 20 kcal/mol. A proof of the consistency in our scheme comes from the results presented above for the amine deprotonation of AMP, MEA, BZA, and also of AMPD [25]: the FEBs turned out to be indistinguishable (about 16 kcal/mol).

We recall (see the Methods Section) that metadynamics relied on a set of collective variables designed to overcome simple geometric constraints and explicitly promote more atoms to participate in the reactions. Depending on the initial configuration of the water molecules comprised between the amine and a CO_2_ molecule, two paths were observed, namely, directly involving either one or two water molecules. Simultaneously with the protonation of the amine, the transfer of the hydroxyl ion occurs either directly to an approaching CO_2_ or to a mediating water molecule. Bending of the COO moiety to accommodate the hydroxyl anion is likely to be an activated process.

In Ref. [41], bicarbonate is considered to form from the simultaneous reactions between the amine, one water molecule, and one CO_2_ molecule. The activation energy for this process was calculated to be 18 and 14.5 kcal/mol for MEA and AMP, respectively. In Ref. [42], the search for a path leading to the formation of the bicarbonate was made in the case of AMP and pursued using free MD run at 400K to “speed up reactions”, for a duration of 120 ps. While the amine protonation was detected, the OH^−^ attack on CO_2_ was not observed.

### 2.4. What Have We Learned?

In this subsection, we briefly summarize the main results presented above.

The most interesting findings of our investigation concern the comparison between MEA and AMP, which we summarize as follows: (i) Our calculations suggest one more possible explanation for the rare observation of carbamates among the products of CO_2_ absorption in AMP solutions: the extremely low probability that the AMP-carbamate forms via a zwitterion. This behavior contrasts with the one we observed in our simulations of MEA and AMPD because the zwitterion was not prone to spontaneously release CO_2_. (ii) Direct detachment of the carboxylate ion from the AMP-carbamate has a much lower free-energy barrier than in MEA and AMPD. This barrier has the same value (within the accuracy of the calculations) as the one corresponding to the CO_2_ release following the carbamate reversion to zwitterion and induced by the deprotonation of a protonated amine (the rate-limiting step). Hence, one could tentatively argue that the AMP carbamate, once formed, has a higher tendency to desorb CO_2_ than the MEA carbamate. Moreover, (iii) our discussion of bicarbonate formation in the presence of amine emphasizes the inadequacy of our and any other available simulation method to account for differences due to the basicity of the amines, as long as their pK_a_ values differ by less than three to four units.

Our simulations of the reactions in the BZA solvent show similar energetics to MEA.

As in our previous publications, we emphasize once more the primary role of water that cannot be reduced to the dielectric effect of a continuum polarisable model. Water molecules act as both proton acceptors and donors, and thus as mediating agents for both proton and hydroxyl ion transfer. Moreover, a strong reorganization of the solvent accompanies and facilitates CO_2_ release.

## 3. Methods

### 3.1. Methods Used in the Present Investigation

We refer the reader to our previous studies of CO_2_ capture in aqueous MEA [24,26] and AMPD [25]—including the Supplementary Materials—for a description of our computational schemes as well as their tests and justification. For a critical review of other simulation studies on MEA and for a critical discussion on the limitations of molecular dynamics applied to the specific systems and chemical reactions of interest, we refer to Ref. [26]. Here, we briefly recall the main characteristics of the methodologies and clarify other details that are specific to the modeling of AMP and BZA (see also the Supplementary Materials).

Our calculations involve the following steps: (i) classical MD, aided with replica exchange, aimed to equilibrate the CO_2_–amine–water systems (at 30 wt.%); (ii) DFT-based (Car–Parrinello) molecular dynamics at the same amine concentration, which corresponds to that typical of experimental investigation and of practical use; and (iii) simulations of the relevant reactions using metadynamics as a sampling-enhancement technique [45,46].

Note that the equilibration process in (i) is used to provide the initial configurations in step (ii) and to identify the most populated amine isomers to be studied further. The relative populations of such isomers in solution are uncorrelated with the energy difference of the isolated molecules (see the Supplementary Materials).

The force fields used in classical MD simulations were chosen according to the protocol introduced by da Silva et al. in Ref. [47] for the MEA–CO_2_–water system. For the amines, the parametrization of the general amber force field (GAFF) [48] was selected, with charges derived from the electrostatic potential calculated within the same DFT scheme (BLYP-D2 exchange–correlation function [49,50,51]) used in our DFT-based MD. The force constants were further adjusted to reproduce the frequencies in the stretching modes of the amine molecules evaluated using the DFT BLYP-D2 scheme. The modified TIP3P model in Ref. [52] was used for water and the modified EMP2 potential proposed in Ref. [47] was used for CO_2_.

DFT calculations were performed using the BLYP-D2 approximation for the exchange–correlation function. Prior to MD simulations, calculations for the amines in the gas phase were performed, which allowed for a comparison of structural properties with the predictions of a related hybrid function (B3LYP [53,54]). Some results are given in the Supplementary Materials.

In the case of MEA solutions, we studied a few systems to detect possible effects of the model size as well as the effects of temperature on the results [24]. Given the minor differences found in the physical behavior of the various samples, simulations of AMPD solutions were only performed at room temperature and for models of an intermediate size comparable with the one used for MEA solutions [25]. The same applies to the simulations we present here for AMP and BZA.

The key simulations performed in the present study used metadynamics-driven MD [45,46]. As in our reference calculations for MEA and AMPD, simple geometric parameters were used as collective variables for the reactions related to the formation and dissociation of carbamate, namely, the C-N distance for the approach of a CO_2_ molecule to an amine and its detachment from a carbamate, and the N-H distance for deprotonation. On the other hand, a simulation leading to the bicarbonate formation needed a more complex set of collective variables. We chose the one introduced in [55], which is based on patterns of coordination numbers between (selected or all) solute and solvent atoms.

The main advantage of metadynamics-driven MD is that, in contrast with traditional approaches, the final state (the product) of the reaction is not prearranged. In this way, unforeseen reaction paths can also be explored, and unforeseen products can be obtained. Another interesting feature of metadynamics concerns the (Helmholtz) free energy landscape, for which an approximate evaluation can be derived from the bias potential. In particular, one can estimate the free energy barriers (FEBs) involved in specific reactions. If a single collective variable is used, the FES reduces to a simple profile. In all cases, it did not present any noticeable features, such as a local minimum corresponding to an intermediate. Note that all the FEB values reported in the following sections of this paper represent an overestimate because they refer to the bias potential accumulated up to the occurrence of the targeted reaction, without attaining the regime of multiple forward and backward transitions.

**Figure 5:**
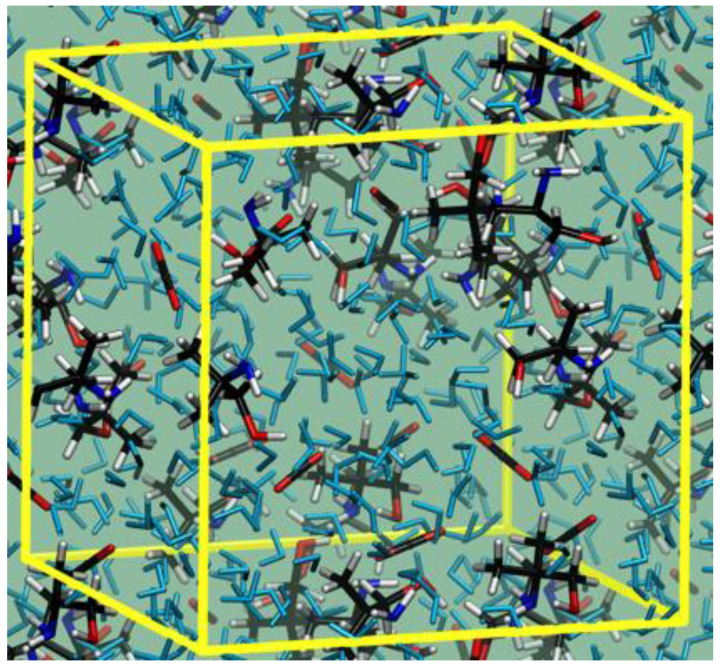
Snapshot showing a sample of aqueous AMP with solvated CO_2_ molecules. Lines define the periodically repeated cell.

The models used to represent the amine solutions with solvated CO_2_ molecules contain 10 amine units, 7 CO_2_ molecules, and 115 water molecules for AMP (see Figure 5) and 10 amine units, 3 CO_2_ molecules, and 139 water molecules for BZA.

### 3.2. Methods Used by Other Authors

Several theoretical approaches have been applied to study the capture of CO_2_ in sterically hindered amines—especially AMP—and compared to MEA. We will mention the results of a few recent papers and compare them with ours. Here, we give a brief account of the methods used in each of the papers.

In Ref. [41], the model consists of a single molecule—be it an amine or a product of its interaction with CO_2_ (both the carbamate–zwitterion and bicarbonate)—and water is represented as a continuum using the SMD solvation model [56]. Geometry optimization is performed at the MP2 level of theory.

In Ref. [42], two different quantum mechanical methods are used: (i) static DFT-B3LYP calculations combined with the SMD model for water and (ii) DFT-based molecular dynamics with a different approximation for the exchange–correlation function (PBE). Method (i) is applied to single molecules (amine, carbamate, bicarbonate) to calculate enthalpy and Gibbs free energy differences, within the standard approximations consistent with the use of SMD (for the gas-phase molecules plus corrections for the solvation energy). Method (ii) is applied to a (periodically repeated) small system including 1–2 amines or their derivatives or bicarbonate and 30 or 16 water molecules to represent the solvent in the case of either AMP or MEA. This model is used to calculate average (total) energy differences, taken over five sampled configurations of the amine + CO_2_ system and the carbamate or the bicarbonate at 313 K.

In Ref. [40], the model consists of a single molecule—amine or amine derivative—and water is represented using the SMD solvation model. For each molecule, geometry optimization is obtained at the MP2 level, and single-point energy calculations are performed within the coupled cluster scheme (CCSD). The relative stability of an amine and the products of its interaction with CO_2_ (carbamate and zwitterion) is established using an estimate of Gibbs free energy differences in analogy with what we mentioned above for Ref. [42].

## 4. Conclusions

In this section, we consider a possible answer to the title of this paper and give an outlook for possible improvements of molecular simulations in the context of carbon capture and release in amine solutions.

Our work contributes to the knowledge of reaction mechanisms that cannot be easily observed experimentally. While this type of information is considered to be relevant for progress in the search for an optimum solvent, it is important to point out that the currently available computational methods for molecular simulations are unable to provide a complete or, especially, a quantitative characterization of the various relevant processes [27]. This conclusion also emerged in the present work. While it was possible to detect interesting unforeseen discrepancies (AMP-MEA-AMPD) and similarities (BZA-MEA) in the reaction dynamics of the investigated amines, quantitative differences must be considered with caution. The representation of the interatomic interactions via non-empirical approaches is important as for any study of chemical reactions. There is no doubt that non-empirical approaches may require more accurate and at the same time more efficient computational schemes than those currently available. However, major limitations currently lie in the unaffordable computational cost of an exhaustive statistical sampling of the configurational space [57,58]. This issue is intrinsic to all simulations of reactions in solution, especially for heterogeneous and multimolecular systems such as those studied here.

With the aim to provide an outlook on the (hopefully) near future of molecular simulations in this field of interest, we add a few more comments:

1. Current molecular simulations necessarily refer to simplified scenarios, thus ignoring the role of several parameters such as the pH of the solution, the CO_2_ concentration, and the operating conditions, which are bound to influence the “performance” of the real systems.
2. So far, most calculations have targeted the absorption of CO_2_ in the solvent and its release. We believe that, in order to better contribute to the tremendous effort to optimize the capturing agents of CO_2_, even staying with amine-based solvents, the focus of molecular simulations should move to physico-chemical processes leading to instability of the solvents and their consequences, e.g., thermal and oxidative degradation, precipitation, and corrosion.
3. Finally, the role of molecular simulations could be enhanced by combining them with experimental approaches, especially with integration into advanced methodologies for process optimization (see, e.g., [59,60]), including artificial intelligence strategies [61,62].

## Figures and Tables

**Table 1:** AMP: Dihedral angles (in degrees) of the three conformers in Figure 2: in the isolated molecules and in the aqueous solutions at room temperature. Standard deviations are given in square brackets.

Dihedral Angle	AMP-a		AMP-b		AMP-c	
	mol	aq	mol	aq	mol	aq
φ (OCCN)	61	64 [8]	54	62 [9]	56	57 [11]
φ (HOCC)	179	171 [9]	−72	65 [15]	42	46 [15]

**Table 2:** BZA: Dihedral angles (in degrees) of both conformers in Figure 3: in the isolated molecules and in the aqueous solutions at room temperature. Standard deviations are given in square brackets.

Dihedral Angle	BZA-a		BZA-b	
	mol	aq	mol	aq
φ (H_1_NCC)	−63	−63 [17]	−60	−63 [17]
φ (H_2_NCC)	177	165 [17]	59	50 [11]
φ (NCCC)	130	138 [9]	3	11 [7]
φ (NCCC)	−50	−58 [11]	−178	−169 [10]

**Table 3:** Number *N* and length *L* of hydrogen bonds between carbamate and water molecules. These values are averaged over 15ps free MD. Standard deviations are given in square brackets.

	*N*	*L* (Å)		*N*	*L* (Å)
AMP-a	3.0 [0.8]	1.82 [0.18]	MEA-a	4.6 [1.2]	1.76 [0.18]
AMP-b	2.7 [0.7]	1.88 [0.18]	MEA-b	4.2 [0.6]	1.77 [0.20]
AMP-c	2.5 [0.9]	1.97 [0.15]			
AMPD-a	4.5 [0.9]	1.84 [0.20]	AMPD-b	4.5 [0.9]	1.84 [0.20]
BZA-a	5.2 [1.1]	1.86 [0.21]	BZA-b	4.8 [1.2]	1.81 [0.19]

**Table 4:** Shortest length (in Å) of the H bond paths between the nitrogen in the zwitterion and the nitrogen in the other amine moieties (N(amine)) or an oxygen in the carboxylate anion (O(COO)). Calculations refer to both BZA conformers (see text). Results for MEA and AMPD conformers are from Ref. [25], see the Supplementary Materials. Standard deviation is given in square brackets.

	BZA		MEA		AMPD	
	(a)	(b)	(a)	(b)	(a)	(b)
N (amine)	4.2 [0.9]	4.2 [0.7]	3.3 [0.8]	3.7 [1.0]	5.3 [0.7]	5.6 [1.4]
O (COO)	7.2 [0.8]	6.5 [0.6]	5.3 [0.7]	4.9 [1.0]	6.6 [1.6]	6.5 [1.6]

## Data Availability

Not applicable.

## Supplementary Materials

The following supporting information can be downloaded at: https://www.mdpi.com/article/10.3390/molecules28186447/s1, Other details of the computational setup; structural properties of the isolated molecules calculated in different schemes: AMP (Table S1) and BZA (Table S2); and images of the carbamate isomers for AMP (Figure S1) and BZA (Figure S2). References [63,64,65,66] are cited in the Supplementary Materials.
