# Supplementary material for: CO2 Capture and Release in Amine Solutions: To What Extent Can Molecular Simulations Help Understand the Trends?
Source: Molecules. 2023 Sep 5;28(18):6447. doi: 10.3390/molecules28186447 (PMC10534568; doi:10.3390/molecules28186447)
Supplement: Supplementary file 1 [file molecules-28-06447-s001.zip › molecules-2523850-supplementary.pdf]

Capture and Release of CO_2 from Amine Aqueous Solutions: To What Extent Can Molecular Simulations Help Understand the Trends?

Supplementary Material

Changru Ma[1, +]; Fabio Pietrucci[2]; Wanda Andreoni [1,*]
1. Institute of Physics, Ecole Polytechnique Federale de Lausanne, Lausanne, Switzerland; 2. Sorbonne Université, Muséum National d'Histoire Naturelle, UMR CNRS 7590, IRD, Institut de Minéralogie, de Physique des Matériaux et de Cosmochimie, IMPMC, F-75005 Paris, France
*Corresponding author
+ Present Address: Eco-Efficient Products and Processes Laboratory, UMI 3464 CNRS/Solvay 3966 Jin DuRoad, Xinzhuang, Industrial Zone, Shanghai 201108, China

Methods: For the details of the calculations, the main text also refers to our previous publications, including the Supplementary Materials sections. The software used includes LAMMPS[1] for classical molecular dynamics, CPMD[2] for *ab initio* MD, and the PLUMED[3] library for metadynamics-driven molecular dynamics.

Classical molecular dynamics equilibration runs were performed for the amine solution at 300 K, first at constant pressure (about 5ns) to control the density at zero pressure, and then for 10 ns at constant volume. These runs were followed by replica-exchange runs were made in the range 300-600K for a duration of 160ns.

In the ab initio Car-Parrinello MD, the valence electron wavefunctions are expanded in plane waves with 80Ry cutoff and the core-valence interaction is represented by well-established psudopotentials [4]. Hydrogen atoms were replaced by deuterium. The systems here simulated are contained in periodically repeated cubic box, of edge 17.52A and 18.38A for AMP and BZA, respectively.

In the metadynamics runs: Gaussians of standard deviation 0.10Å and height 1.15 kcal/mol were added every 0.2 ps.

Estimates of the enthalpic contributions to the free-energy barriers are obtained from averaging the total energy over 50 equally-spaced configurations of the free-MD trajectories at the reactant, transition and product states of the reactions (15ps each).

In the simulations of the bicarbonate formation, the coordination pattern considered in the CVs involved two water molecules, CO_2 and the functional group of the amine.

Tables and Figures: The definition adopted for a hydrogen bond: O-O or O-N distance cutoff = distances 3Å; angle <OHO or <OHN angle cutoff = 20˚.

Table S1. AMP: Three conformers as in Figure 3 of the main text. Selected structural properties of the isolated molecules calculated within different schemes: BLYP-D2(B2) [5-7] and B3LYP(B3) [8,9] exchange-correlation functionals. P stands for pseudopotential; A for all-electron. Bond-lengths are in *Å;* bond and dihedral angles in degrees. Note that (a) is the lowest energy isomer. In the scheme of the MD simulations (BLYP) (b) and (c) are higher by 4&5kcal/mol.

	AMP-a			AMP-b			AMP-c		
	B2P	B2A	B3A	B2P	B2A	B3A	B2P	B2A	B3A
N-C	1.48	1.48	1.47	1.48	1.49	1.48	1.49	1.49	1.48
C-O.	1.45	1.44	1.42	1.45	1.45	1.42	1.44	1.44	1.42
<NCC(CH_2)	112	112	112	107	107	106	106	106	106
<NCC(CH_3)	108	109	108	109-112	108-113		108-113		
φ (OCCN).	61	64	61	54	53	54	56	56	54
φ (HOCC)	179	179	175	-72	-71	-71	42	43	41

Table S2. BZA: Two conformers as in Figure 4 of the main text. Selected structural properties of the isolated molecules calculated within different schemes: BLYP-D2(B2) and B3LYP(B3) xc functionals. P stands for pseudopotential; A for all-electron. Bond-lengths are in *Å;* bond and dihedral angles in degrees. In the scheme of the MD simulations (BLYP) (a) and (b) are essentially degenerate.

	BZA-a			BZA-b		
	B2P	B2A	B3A	B2P	B2A	B3A
N-C	1.49	1.49	1.47	1.47	1.47	1.40
<NCC	111	111	112	118	118	119
φ (H_1NCC)	-63	-65	-66	-60	-60	-61
φ (H_2NCC)	177	177	174	59	58	60
φ (NCCC)	130	137	140	3	3	2
φ (NCCC)	-50	-44	-41	-178	-178	-178

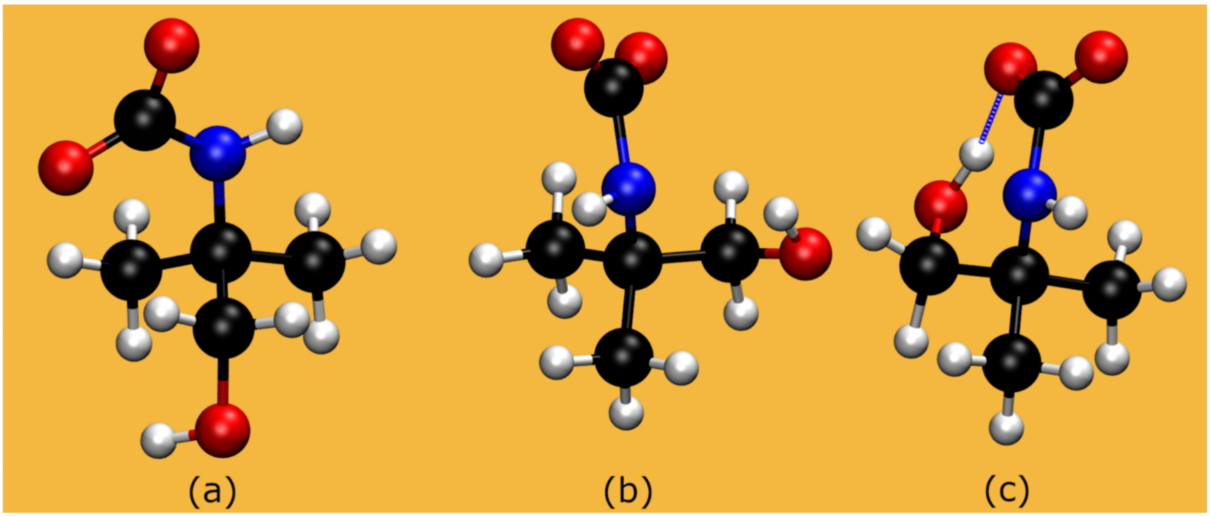

Figure S1. AMP carbamates.

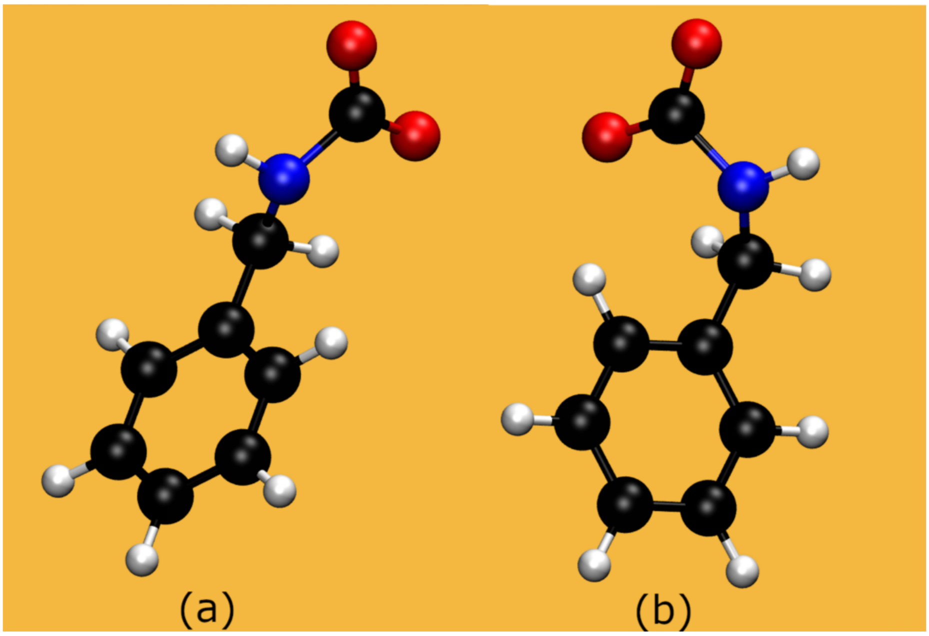

Figure S2. BZA carbamates.

1. Plimpton, S.; Fast Parallel Algorithms for Short-Range Molecular Dynamics. J. Comp. Phys. **1995**, 117, 1-19. https://www.lammps.org/

2. CPMD, IBM Corp 1990-2022 and MPI für Festkörperforschung Stuttgart 1997-2001. https://www.cpmd.org/

3. Bonomi, M.; D. Branduardi, D.; Bussi, G.; Camilloni, C.; Provasi, D.; Raitei, P.; Donadio, D.; Marinelli, F.; Pietrucci, F.; Broglia, R.A.; Parrinello, M. PLUMED: a portable plugin for

free-energy calculations with molecular dynamics. *Comput. Phys. Commun.* **2009**, *180*, 1961-1972 https://www.plumed.org

4. Troullier, N.; Martins, J.L. Efficient pseudopotentials for plane-wave calculations **1991**, *Phys. Rev. B* 43, 1993-2006.

5. Becke, A.D. Density-functional exchange-energy approximation with correct asymptotic behavior. *Phys. Rev. A* **1988**, *38*, 3098-3100

6. Lee, C.; Yang, W.; Parr, R.G.; Development of the Colle-Salvetti correlation-energy formula into a functional of the electron density. *Phys. Rev. B* **1988**, 37, 785-789.

7. Grimme, S. Semiempirical GGA-type density functional constructed with a long-range dispersion correction. *J. Comput. Chem.* **1988**, *27*, 1787-1799.

8. Stephens, P.J., Devlin, F.J., Chabalowski, C.F., Frisch, M.J. Ab Initio Calculation of Vibrational Absorption and Circular Dichroism Spectra Using Density Functional Force Fields. J. Phys. Chem. **1994**, *98*, 11623-11627

9. Vosko, S.H.; Wilk, L.; Nusair, M. Accurate spin-dependent electron liquid correlation energies for local spin density calculations: a critical analysis. *Can. J. Phys.* **1980**, *58*, 1200-1211
